# Mandibular cortical bone structure as risk indicator in fractured and non-fractured 80-year-old men and women

**DOI:** 10.1186/s12903-021-01829-0

**Published:** 2021-09-24

**Authors:** Grethe Jonasson, Azar Hassani-Nejad, Magnus Hakeberg

**Affiliations:** 1 Department of Behavioral and Community Dentistry, Institute of Odontology at the Sahlgrenska Academy, University of Gothenburg, Box 450, 405 30 Gothenburg, Sweden; 2 Research and Development Centre, Sven Eriksonplatsen 4, 50338 Borås, Sweden; 3 Clinic of Oral and Maxillofacial Radiology, Public Dental Service, P.O.Box 7163, SE-402 33SE-402 33163 Gothenburg, Sweden

**Keywords:** Bone, Fracture, Human, Mandible, Osteoporosis, Radiographs

## Abstract

**Objective:**

To investigate the association between mandibular cortex parameters and fracture in a group of 286 men and women, 79–80 years of age.

**Study design:**

In a cross-sectional study, the mandibular cortex was evaluated with Klemetti’s index for cortical erosion. The cortical thickness was measured with a ruler adjusting for the magnification factor. The odds ratio (OR) for fracture when having a severely eroded cortex or a cortex thickness < 3 mm was calculated.

**Results:**

A normal cortex was found in 65% of men, whereas only 7% had a severely eroded cortex. The OR for severely eroded cortex  as fracture risk predictor was significant (2.32; 95% CI 1.3–4.2), also when the female group was evaluated separately. A significant difference was found between the mean thickness for men (3.96 mm) and women (2.92 mm), respectively. The OR for cortical thickness < 3 mm was significant (2.00; 95% CI 1.1–3.6) in the total group, but not when men and women were evaluated separately.

**Conclusions:**

Among old women, the cortical parameters were significantly associated with prevalent fracture. In old men, other circumstances may be more important.

## Introduction

Osteoporotic fractures are associated with an increased risk of future fractures, a high rate of mortality, and considerable medical costs. Many women and men will suffer fragility fractures that could be prevented if those at risk were identified at an early stage. Bone quality and bone strength are not easily defined. They are dependent on bone mass, structure, size, and microstructural features (e.g., collagen fibres, crystal size), but also on bone turnover rate, microdamage and the degree of secondary mineralisation [1].

The osteoporosis diagnosis is based on an assessment of skeletal bone mineral density (BMD) with dual X-ray absorptiometry (DXA). A T-score less than − 2.5 is the WHO definition of osteoporosis. It is closely associated with fragility fractures; nevertheless, 73% of all new fractures the following six years occurred in individuals with normal BMD or osteopenia [2]. A prevalent fracture increased the fracture risk so that women with osteopenia and a prevalent fracture had at least the same risk as women with osteoporosis alone [2]. In another study, up to 96% of new fragility fractures in the next ten-year period occurred in women without osteoporosis at baseline [3]. The low sensitivity of DXA as a fracture predictor is the main reason why population screening is not recommended for women at menopause. Nowadays, other risk variables, with or without BMD, are considered when the fracture risk is assessed; for instance, age, body mass index, a history of fracture, parental history of hip fracture, use of oral glucocorticoids, rheumatoid arthritis, current smoking, and alcohol intake are used to calculate the ten-year fracture probability in the FRAX models [3].

Most adults in Western countries visit their dentists regularly and radiographs are taken. It is therefore possible that general dental practices may be suitable places for identifying the patients most at risk. Cortical bone is well presented on panoramic radiographs and trabecular bone better imaged on periapical radiographs. In a new article regarding clinical guidelines for use of panoramic radiographs in screening for osteoporosis, Taguchi et al. 2021 stated that postmenopausal women with a severely eroded cortex have an increased risk of having low skeletal BMD, as well as fragility fractures [4], whereas women with a mandibular cortical width of < 3 mm may be at risk of having low skeletal bone mineral density (BMD) but not fragility fractures [4].

Several research groups have demonstrated use of panoramic radiographs as predictors of low BMD. Few have used them as fracture predictors and there is still a need to test them as markers of fracture risk. Because we have a sample of old individuals with fracture data, the aim of the present investigation was to test cortical erosion and thickness as fracture predictors in 80-year-old men and women.

## Methods

### Subjects

Of 662 participants in a medical study of elderly men and women in Gothenburg, 286 individuals (129 men and 157 women) consented to dental radiography. All were born in 1930 (age at examination, 79–80 years old). One hundred and seventy of these individuals had participated in previous prospective population studies in Gothenburg, the Gerontological and Geriatric Population Study in Gothenburg, Sweden (H70), and the Prospective Population Study of Women in Gothenburg, Sweden. One hundred sixteen participants had not been examined previously.

All subjects completed a questionnaire concerning fracture and medical history. The fractures considered were self-reported and sustained anterior to the panoramic radiograph. Seven (2.5%) patients had medication with corticosteroids; 40 (14%) had calcium supplements and 18 (6.3%) had bisphosphonate medication.

The Central Ethical Review Board, University of Gothenburg, Göteborg, Sweden, approved the study (Reg. No. 075-09, T257-09). The examinations comply with the Declaration of Helsinki, and the participants gave their informed consent.

### Digital radiographs

All digital panoramic radiographs were taken at the Department of Oral and Maxillofacial Radiology, Institute of Odontology, The Sahlgrenska Academy, University of Gothenburg. The panoramic radiographs were taken using a Scanora (Orion Soredex, Helsinki, Finland) at 66–70 kV and 20 mA. The radiographic exposure factors were adjusted according to the size of each participant. Larger persons were exposed with higher kV and mA than smaller ones.

The images were archived in RIS (radiographic information system) and PACS (picture archiving communications system). The images were evaluated on a workstation equipped with a DELL computer (Optiplex 755/780 MT and Precision T3400, DELL AB, Stockholm, Sweden) with a graphics card (NVIDIA GeForce 8600 Series GPU 32-bit, Matrox MED2mp-DVI) and a 20-in. flat panel monitor. All evaluations were performed on a monochromatic screen (OLÓRIN Medic Line ML 207D TFT-LCD, Olorin AB, Kungsbacka, Sweden) with a resolution of 1280 × 1024 pixels.

### Intra-observer and interobserver agreement

Cortical erosion was assessed by three dentists, one experienced in classifying patterns in oral radiographs and two oral and maxillofacial radiologists. Before starting the assessments, the observers met several times for calibration and discussions. Fifty panoramic radiographs were evaluated twice at four-week intervals. The intra-individual and inter-individual agreement regarding the assessment of the mandibular cortical index, i.e., compact bone erosion, was calculated using weighted Kappa statistics. The Kappa index was interpreted according to Landis and Kock [5]. Cortical thickness was measured twice on 20 radiographs, two years apart, and correlated to each other.

### The mandibular cortical index (MCI) and cortical thickness

**Fig. 1:**
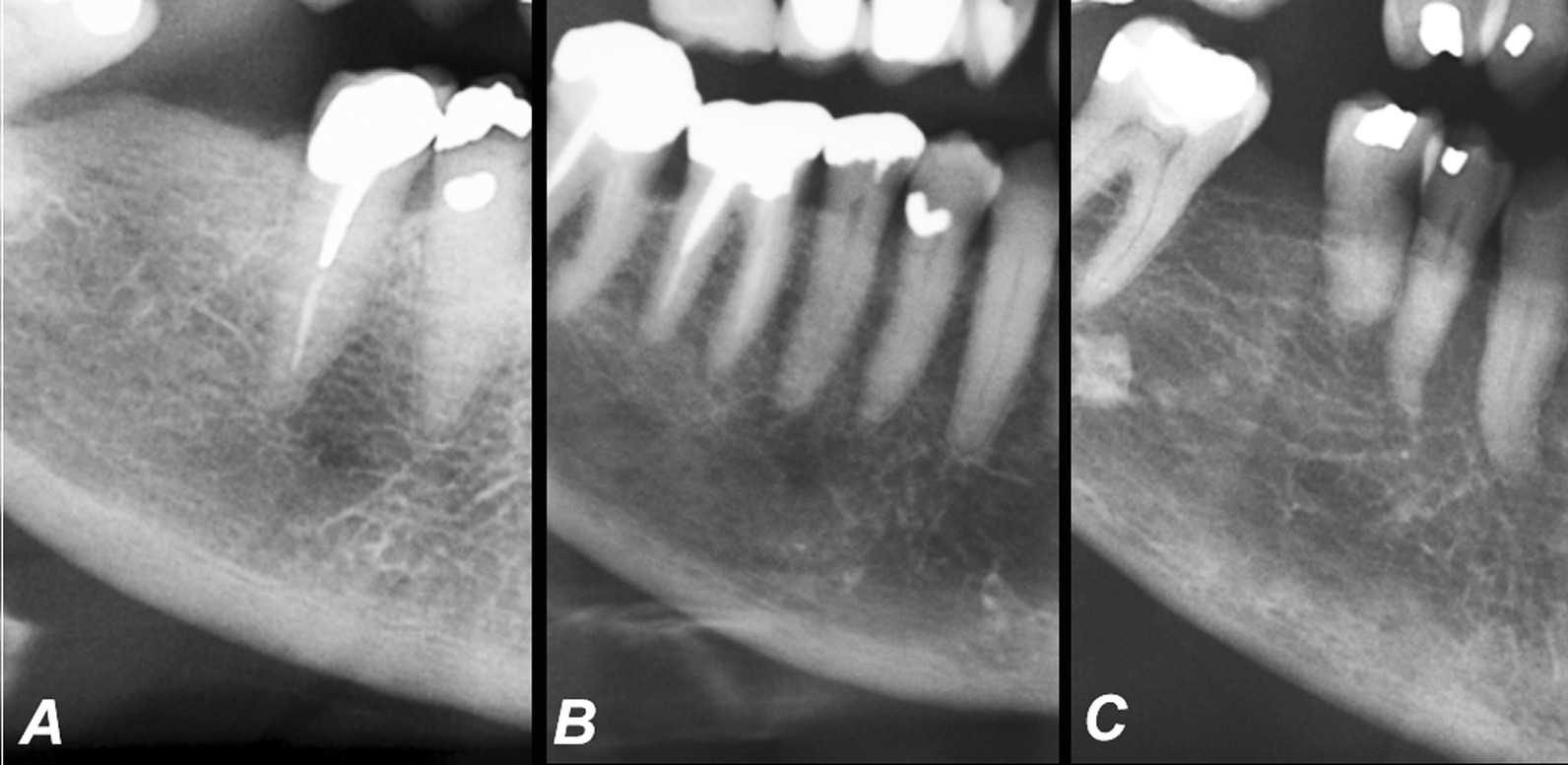
Reference panoramic radiographs for mandibular cortical index (MCI). **a** A normal cortex (MCI-1) with an even endosteal margin. **b** A moderately eroded cortex (MCI-2) with semilunar defects. **c** A severely eroded cortex (MCI-3) with heavy endosteal cortical porosities

Cortical bone lying distal to the mental foramen was categorized into three groups, Fig. 1 [6–10].

**Fig. 2:**
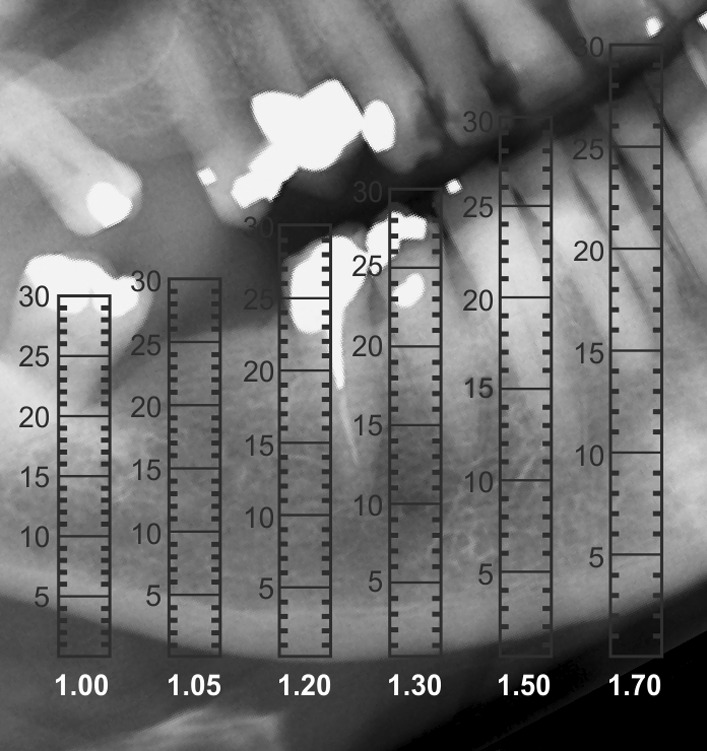
Measurement of cortical thickness on panoramic radiographs with a transparent ruler that takes into account the magnification factor (1.3)

The cortical thickness was measured using the “natural size” of the digital panoramic radiograph and a specially developed transparent ruler that took the magnification of the panoramic radiograph into account (magnification factor for all panoramic radiographs was 1.3), Fig. 2.

The thickness was measured slightly distally of the mental foramen as a small area (1–3 mm) of the cortex just below the foramen is often clearly thicker than the rest of the distal cortex. This small area corresponds to the phenomenon called trabecular bone tail connected to the cortical bone [4, 10].

### Statistical methods

The analysis of variance (ANOVA) for continuous variables and the Kruskal–Wallis non-parametric test for ordinal variables were used for significance testing between groups. Bivariate logistic regression analyses were used to calculate the odds ratio for fracture using following predictors: cortical erosion, cortical thickness, bisphosphonate, calcium, corticosteroid, BMI or sex). Similarly, multiple logistic regression analyses were performed to calculate the odds ratio for fracture when using cortical parameters and including bisphosphonate medication. The cortical parameters were transformed to dummy variables. These analyses were performed using Epi Info version 3.5 (Centers for Disease Control and Prevention, Atlanta, GA). The Relative Risk (RR) was calculated using DJR Hutchon’s calculator (www.hutchon.net/ConfidRR.htm). Stata version 13.1 (StataCorp, College Station, TX, USA) was used for calculations of predictive power (sensitivity, specificity, positive and negative predictive values (PPV and NPV), and area under curve (AUC).

## Results

The mean height was 161.9 ± 5.7 cm (range 145–177 cm) for women and 175.7 ± 6.6 cm (range 156–195 cm; p < 0.0001) for men. The mean BMI for women was 25.7 ± 4.3 (range 18–39) and 26.2 ± 3.2 (range 19–36; p > 0.05) for men.

### Non-responding individuals

No differences in fracture prevalence and medication were found between the 286 included men and women and those without dental radiographs (n = 376). However, the 376 non-responders had fallen more often (mean no. of falls 0.93 vs 0.63; p = 0.019) and hurt themselves significantly more frequently than the 286 responders (0.94 vs 0.57; p = 0.0009). Furthermore, they had had rheumatic disease for a longer time than the responders (1.64 years vs 1.23 years; p = 0.008).

### Intra-observer and interobserver agreement

The interobserver agreement for cortical erosion was moderate to good (0.59–0.79), and the intra-observer agreement was good to very good (0.67–0.91) for the mandibular cortical index. For cortical thickness, the intra-observer agreement, expressed as the correlation between two measurements, was highly correlated (r = 0.88; p < 0.001, as was the interobserver agreement (r = 0.85; p < 0.001).

### Fracture

Seventy individuals in the examined group reported previous fractures in adulthood at sites typically of fragility fractures; i.e., fracture rate was 24.5%. If the fracture was obviously caused by an accident it was excluded, but otherwise, no attempt was made to distinguish between fragility and traumatic fractures. Forty-nine women and 21 men had sustained one or more fractures; i.e., a fracture rate of 31.2% in women and 16.3% in men (p = 0.0035). Four individuals had sustained three fractures and twelve individuals had had two fractures. In all analyses, fractures are counted only once for each individual, meaning that the individuals were categorized as fractured or non-fractured. Of 90 fractures in 70 individuals, hand fracture represented 57.8% of all fractures (73% of them in women; p = 0.003), spine fracture represented 15.5% (64.3% in women; p > 0.05), hip fracture represented 13.3% (91.7% in women; p = 0.03), upper-arm fracture represented 10% (66.7% in women; p > 0.05), and pelvis fractures represented 4.4% and were only found in women.

### The mandibular cortical index (MCI) and cortical thickness

In the total group of men and women, 124 (43.4%) had a normal cortex, 89 (31.1%) had a moderately eroded cortex, and 73 (25.5%) had a severely eroded cortex. The difference in cortical erosion degree between men and women was significant (p < 0.0001). A normal cortex was found in 65.1% of the men, and only 7.0% of men had a severely eroded cortex. Among the women, 25.5% had a normal cortex and 40.8% had a severely eroded cortex. This means that 67% of those with a normal cortex were men and 88% of those with a severely eroded cortex were women. Nine men had a severely eroded cortex and one of them was fractured. Sixty-four women had a severely eroded cortex and 26 of them had a fracture. Sex, degree of erosion and cortical thickness in mm in fractured men and women are presented in Table 1.

**Table 1:** Sex, degree of erosion (MCI-3 = most eroded) and cortex thickness in mm in fractured men and women

	Total (70 of 286)	Men (21 of 129)	Women (49 of 157)
Fractured	70	21	49
*Cortical erosion*			
MCI-1 (%)	22 (31.4)	10 (47.6)	12 (24.5)
MCI-2 (%)	21 (30)	10 (47.6)	11 (22.5)
MCI-3 (%)	27 (38.6)	1 (4.8)	26 (53)
*Cortical thickness (mm)*^a^			
1	4 (5.7)	0	4 (8.2)
2	21 (30)	1 (4.8)	20 (40.8)
3	14 (20)	3 (14.3)	11 (22.4)
4	20 (28.6)	10 (47.6)	10 (20.49
5	10 (14.3)	6 (28.6)	4 (8.2)
6	1 (1.4)	1 (4.8)	0

^a^Measurements were performed with an accuracy of one decimal

A significant difference in erosion degree was found between individuals with and without fracture (total group; p = 0.003). The odds ratio for a severely eroded cortex as a predictor of fracture was significant (OR = 2.32 (95% CI 1.3–4.2; p = 0.0045). The relative risk was 1.8 (95% CI 1.2–2.7). However, when the male group was analysed separately, no significant difference was found between the fracture and the non-fracture group (p > 0.05), whereas the difference remained significant for the female group (OR = 2.14 (95% CI 1.1–4.3; p = 0.03). For cortical erosion as a fracture predictor, the sensitivity was 38.5%, the specificity 78.7%, PPV 36.7%, NPV 79.9%, and the AUC 58.6% (95% CI 52.2–65.2%).

The mean cortical thickness was 3.4 ± 1.2 mm (range 1–6 mm). A significant difference was found between the mean thickness for men (4.0 ± 1.0, range 2–6 mm) and women (2.9 ± 1.1 mm, range 1–6 mm; p < 0.0001). Only ten men had a cortical thickness < 3 mm, one of them was fractured. Sixty-two women had a cortex thickness less than 3 mm and 24 of them were fractured. In a logistic regression analysis with a recommended risk factor set to < 3 mm cortical thickness, the odds ratio for fracture was significant (OR = 2.0; 95% CI: 1.1–3.6; p = 0.02). When separate analyses were performed for the male and female groups, the ORs for having fracture when cortical thickness was less than 3 mm, were no longer significant (OR = 1.82 (95% CI 0.9–3.6; p = 0.09). The RR for cortical thickness less than 3 mm as fracture predictor for the total group was significant (1.6; 95% CI 1.1–2.4).

For cortex thickness as a fracture predictor, the sensitivity was 35.8%, the specificity 78.2%, PPV 34.7%, NPV 79.0%, and the AUC 57.0% (95% CI 50.6–63.0%). Both bisphosphonate and calcium were associated with fracture, but corticosteroid medication and body mass index (BMI) was not (Table 2).

**Table 2:** Eight bivariate logistic regressions analyses for calculating odds ratio for fracture predictors, and two multiple logistic regression analyses

Models	OR (95% CI)	Coefficient	*P*
I. Fracture/severe cort. erosion	2.32 (1.3–4.2)	0.846	0.0045
II. Fracture/thickness < 3 mm	2.00 (1.1–3.6)	0.692	0.0206
III. Fracture/bisphosphonate	5.57 (2.1–15.0)	1.717	0.0007
IV. Fracture/calcium	3.92 (2.0–7.8)	1.366	0.0001
VI. Fracture/corticosteroid	0 (0–> 10^12^)	− 12.489	0.9704
VII. Fracture/BMI	0.93 (0.9–1.0)	− 0.073	0.0516
VIII. Fracture/sex	2.36 (1.3–4.2)	0.857	0.0036
I. Fracture/erosion/bisphos			
Severe cortical erosion	1.95 (1.1–3.6)	0.670	0.0304
Bisphosphonate medication	4.46 (1.6–12.3)	1.495	0.0040
II. Fracture/thickness/bisphos			
Cortical thickness < 3 mm	1.65 (0.9–3.1)	0.502	0.1101
Bisphosphonate medication	4.68 (1.7–12.9)	1.543	0.0029

Eight fracture risk factors were tested: severely eroded cortex with heavy endosteal cortical porosities (Fig. 1), a cortical thickness < 3 mm (Fig. 2), bisphosphonate medication, calcium, corticosteroid, BMI and sex

## Discussion

In the present investigation, significant differences were found between men and women regarding cortical thickness and erosion. A normal cortex (MCI1) was found in 65% of the men and 26% of the women, whereas 41% of the women but only 7% of the men had a severely eroded cortex. The odds ratios were significant for both cortical thickness < 3 mm and for severely eroded cortex as fracture predictors in the total group, and for severely eroded cortex in the separate female group. There were few men in the risk groups, which may explain the lack of significance. We noticed that in these old individuals, a severely eroded cortex goes together with a thin cortex and with a sparse trabecular pattern in the alveolar process.

Cross-sectional studies have shown that certain alterations of the mandibular trabecular pattern are associated with the fracture rate [11], and a moderately and severely eroded cortex plus a thinner cortex can be related to self-reported fracture in women above 60 years of age [12]. In longitudinal studies, a sparse trabecular pattern and cortical erosion in the mandible were predictors of a future incident fracture [13, 14]. Sparse trabeculation was found to be a serious risk factor identifying middle-aged women at high risk of future fractures many years before the first fracture occurred, whereas cortical erosion was useful as a predictor of fractures later in life [14]. In general, the use of clinical risk factors improves the sensitivity of the tests without decreasing the specificity [11]. Likewise, mandibular sparse trabeculation and severely eroded cortices have a substantial additive effect on the fracture prediction by FRAX [15, 16], but cortical thickness did not [16]. In a cross-sectional study using digital intraoral radiographs from 80-year-old men and women, it was found that few men had a sparse trabecular pattern, but if they did, the odds ratio for fracture was high (OR = 5.5), even higher than for women (OR = 3.4) [17].

In the mentioned longitudinal study of women [14], the percentage of women with a severely eroded cortex increased with age from 3% at baseline (mean age 44.3 years) to 54% 24 years later, which is not far from the results in the present investigation. The lower value here (41%) may be due to an “age bias”. The present examination period was a very cold and snowy winter with icy streets and many fractures in the community, why it was probably the healthiest 80-year-old individuals who had their panoramic radiographs taken. This also explains why only 286 men and women could be included.

The small number of participants is the main limitation of the study. Very few men were found in the risk groups. This is explained by Ulm et al., who reported that in men, the cortex may increase in thickness with aging by periosteal bone apposition, but this is rarely seen in women [18]. Seeman [19] also found that men gain more periosteal bone than women. Another limitation is the lack of BMD measurements. All 80-year-olds should receive a DXA measurement but, again, the severe winter was an obstacle to participation. Only 58 individuals were measured. However, it is a strength of the study that fracture is the main outcome, as it is rare with fracture studies in old individuals, and the aim of most studies, especially those regarding cortical thickness, is to find alternative ways to diagnose osteoporosis [7, 8, 20–24].

Fracture often happens after trauma, meaning by chance, and is therefore not easily predicted; hence, the values for odds ratios, sensitivity and the Area Under Curve (AUC) are lower than when diagnosing osteoporosis. We did not have the possibility to examine the incidence of fracture after the examination day but used the number of experienced past fractures, which is a limitation. We measured the cortical thickness manually and did not use automatic tools for cortical thickness, but the ruler that compensated for the magnification factor was easy and reliable to use, giving good intra-observer agreement.

Using the same ruler, we had previously found that the cortical thickness increased slightly until the age of 50 and then decreased significantly in parallel with changes in menopausal status [14]. In the group of 78-year-old women, the cortical thickness was 2.3 mm [14], to be compared with the 2.9 mm of 80-year-old women in the present study.

Our results are in contrast with those of Yamada et al. [25]. They found that individuals with eroded mandibular cortices tended to be at increased risk of osteoporosis but not of fractures [25]. The difference between the studies can be attributed to a much younger mean age and to the very small number of fractures in the Japanese study. Okabe et al. [26], investigated a group of 80-year-olds, 262 men and 397 women. They found significant correlations between heel bone density and cortical thickness and cortical erosion, but no significant associations between cortical measurements and the occurrence of fractures within five years of the baseline examination [26]. The difference may be that they used fracture incidence, whereas we used fracture prevalence. The reliability of self-reported information may be a problem because errors can be introduced [27], but also national fracture registries have flaws. Optimally, self-reported fractures should be checked against fracture registries, but it was not possible at the time.

Visual evaluation is operator-dependent, and it is recommended to develop automatic measurements. Different tools have been developed for measurements of cortical thickness [22–24]. Recently, a tool was presented for evaluation of cortical erosion [28].

Many investigations combine oral measurements with clinical variables [15, 16, 20, 29, 30]. Age is an important parameter, and the older the individuals, the better the predictions. High body mass index is protective, whereas a history of fracture, glaucoma, smoking, negative attitude, and the presence of medical conditions may increase the prevalence of osteoporotic fractures [31]. Tooth loss is associated with cortical erosion [32] and osteoporosis [33, 34].

Zebase et al. [35], found that cortical porosity in the radius was associated with fractures in the presence of deteriorated trabecular density, but not if trabecular deterioration was absent. Trabecular density was associated with fractures in the presence of high cortical porosity but not in its absence [35]. These findings may raise the question, whether the cortical parameters should be combined with an evaluation of the trabecular pattern when used for fracture prediction.

Training is important for all indices. Taguchi et al. found that the intra-observer agreement for cortical erosion was significantly increased in observers who specialized in oral radiology [9]. It is easier to assess the cortex than the trabecular pattern on panoramic radiographs. Some trabecular bone structures are not centered in the focal trough and may be less sharp or “lost” [36]. In Sweden, panoramic equipment is mostly found in clinics for specialists, whereas all dental clinics can take periapical radiographs, and these are therefore recommended for evaluation of trabecular bone. Periapical radiographs expose the trabecular bone well, and since bone loss starts in the trabecular compartment, bone changes are first seen here, which is demonstrated in a longitudinal study over 24 years [14].

Hvas et al. [37] raised the question of whether it is ethically justifiable to screen healthy people for osteoporosis. In their qualitative interview study, they found that “awareness of osteoporosis risk caused a feeling of uncertainty and worry in some women. Only women reacting in this way seemed to act to prevent future fractures” [37]. Likewise, it is discussed whether a high incidence of false positive diagnoses of osteoporosis would be unnecessarily stressful for healthy individuals. The choice between high sensitivity or high specificity will probably depend on the health system in the specific country. Taguchi et al. [4] concluded that postmenopausal women with a severely eroded mandibular inferior cortex may have an increased risk of having osteoporosis/fragility fractures. Both Taguchi et al. [4] and Devlin et al. [38] recommend that only those patients with the thinnest mandibular cortices; i.e., 3 mm or less, should be referred for further osteoporosis investigation. In countries like the UK and Sweden, where health care is free or extremely cheap, the workload for primary care is heavy, and a high specificity is thus warranted for dentists involved in identifying individuals at risk, thereby avoiding unnecessary referrals to physicians. The higher specificity motivated the choice of our risk predictors: thickness less than 3 mm and a severely eroded cortex.

## Conclusion

The cortical parameters can be used as markers of fracture risk among old women. Large longitudinal studies are needed before it can be determined whether the same cortical parameters can be used for old men, or if other circumstances are more important.

## Data Availability

The datasets generated during and/or analysed during the current study are available from the corresponding author on reasonable request.
